# Promoting mental health in children and adolescents through digital technology: a systematic review and meta-analysis

**DOI:** 10.3389/fpsyg.2024.1356554

**Published:** 2024-03-12

**Authors:** Tianjiao Chen, Jingyi Ou, Gege Li, Heng Luo

**Affiliations:** Faculty of Artificial Intelligence in Education, Central China Normal University, Wuhan, China

**Keywords:** children and adolescents, digital technology, systematic literature review, meta-analysis, mental health issues

## Abstract

**Background:**

The increasing prevalence of mental health issues among children and adolescents has prompted a growing number of researchers and practitioners to explore digital technology interventions, which offer convenience, diversity, and proven effectiveness in addressing such problems. However, the existing literature reveals a significant gap in comprehensive reviews that consolidate findings and discuss the potential of digital technologies in enhancing mental health.

**Methods:**

To clarify the latest research progress on digital technology to promote mental health in the past decade (2013–2023), we conducted two studies: a systematic review and meta-analysis. The systematic review is based on 59 empirical studies identified from three screening phases, with basic information, types of technologies, types of mental health issues as key points of analysis for synthesis and comparison. The meta-analysis is conducted with 10 qualified experimental studies to determine the overall effect size of digital technology interventions and possible moderating factors.

**Results:**

The results revealed that (1) there is an upward trend in relevant research, comprising mostly experimental and quasi-experimental designs; (2) the common mental health issues include depression, anxiety, bullying, lack of social emotional competence, and mental issues related to COVID-19; (3) among the various technological interventions, mobile applications (apps) have been used most frequently in the diagnosis and treatment of mental issues, followed by virtual reality, serious games, and telemedicine services; and (4) the meta-analysis results indicated that digital technology interventions have a moderate and significant effect size (*g* = 0.43) for promoting mental health.

**Conclusion:**

Based on these findings, this study provides guidance for future practice and research on the promotion of adolescent mental health through digital technology.

**Systematic review registration:**

https://inplasy.com/inplasy-2023-12-0004/, doi: 10.37766/inplasy2023.12.0004.

## 1 Introduction

In recent years, the mental health status of children and adolescents (6–18 years old) has been a matter of wide societal concern. The World Health Organization noted that one in seven adolescents suffers from mental issues, accounting for 13% of the global burden of disease in this age group (World Health Organization, 2021). In particular, the emergence of COVID-19 has led to an increase in depression, anxiety, and other psychological symptoms (Jones et al., 2021; Shah et al., 2021). There is thus an urgent need to monitor and diagnose the mental health of teenagers.

The development of digital technology has brought about profound socio-economic changes; it also provides new opportunities for mental health diagnosis and intervention (Goodyear and Armour, 2018; Giovanelli et al., 2020). First, digital technology breaks the constraints of time and space. It not only provides adolescents with mental health services at a distance but also enables real-time behavioral monitoring for the timely acquisition of dynamic data on adolescents’ mental health (Naslund et al., 2017). Second, due to the still-developing stage of mental health resource building, traditional intervention methods may not be able to meet the increasing demand for mental health services among children and adolescents (Villarreal, 2018; Aschbrenner et al., 2019). In addition, as digital natives in the information age, adolescents have the ability to use digital technology proficiently, and social media, such as the internet, has long been integrated into all aspects of adolescents’ lives (Uhlhaas and Torous, 2019). However, it is worth noting that excessive reliance on digital technology (e.g., internet and smartphone addiction) are also common triggers of mental problems among youth (Wacks and Weinstein, 2021). Therefore, we must be aware of the risks posed by digital technology to better utilize it for promoting the mental health of young people.

Mental health, sometimes referred to as psychological health in the literature, encompasses three different perspectives: pathological orientation, positive orientation, and complete orientation (Keyes, 2009). Pathological orientation refers to whether patients exhibit symptoms of mental issues, including internalized mental disorders (e.g., depression and anxiety) and behavioral dysfunctions (e.g., aggression, self-harm) as well as other mental illnesses. Studies have indicated that both internalizing and externalizing disorders belong to different dimensions of mental disorders (Scott et al., 2020), and internalizing symptoms often occur simultaneously with externalizing behaviors (Essau and de la Torre-Luque, 2023). The positive orientation suggests that mental health is a positive mental state, characterized by a person’s ability to fully participate in various activities and to express positive and negative emotions (Kenny et al., 2016). The complete orientation integrates pathological and positive orientation (Antaramian et al., 2010), suggesting that mental health means the absence of mental issues and the presence of subjective well-being (Suldo and Shaffer, 2008). The development of social emotional abilities helps to promote subjective well-being for adolescents during social, emotional, and cognitive development (Cejudo et al., 2019). Adolescents with mental health issues may thus exhibit pathological symptoms or lack of subjective well-being due to a lack of social emotional abilities. In this study, mental health is defined as a psychological state advocated by the complete orientation.

Promoting mental health using digital technology involves providing help through digital tools such as computers, tablets, or phones with internet-based programs (Hollis et al., 2017). Currently, various digital technologies have been tested to address mental health issues in young individuals, including apps, video games, telemedicine, chatbots, and virtual reality (VR). However, the impact of digital technology interventions is affected by various factors (Piers et al., 2023). Efficacy varies based on the kind of mental health issues. Individuals with mental illness related to COVID-19 may profit more from digital interventions than those experiencing depression and anxiety. Moreover, studies reveal that several mental health conditions in young people deteriorate with age, particularly anxiety and suicide attempts (Tang et al., 2019). The impact of digital technology interventions may therefore differ depending on the adolescent’s age. Having psychological problems usually indicates that people are in an unhealthy mental state for a long time, so an enduring intervention may have greater efficacy than a short-term one. Earlier studies have also suggested that the outcomes of treatment are linked to its duration, with patients receiving long-term treatment experiencing better results (Grist et al., 2019).

Although more digital technologies are being used to treat mental health issues, the most important clinical findings have come from strict randomized controlled trials (Mohr et al., 2018). It is still unclear how these interventions affect long-term care or how they would function in real-world settings (Folker et al., 2018). There is much relevant empirical research, but it is scattered, and there is a need for systematic reviews in this area. In previous studies about technology for mental health, Grist et al. (2019) analyzed how digital interventions affect teenagers with depression and anxiety, but their study only considered mental disorders, without considering other mental health issues. Cheng et al. (2019) examined serious games and their application of gamification elements to enhance mental health; however, they overlooked various technological approaches beyond serious games and did not give adequate consideration to the diverse types and features of technology. Eisenstadt et al. (2021) reviewed how mobile apps can help adults between 18 and 45 years of age improve their emotional regulation, mental health, and overall well-being; however, they did not investigate the potential benefits of apps for teenagers.

The present study reviews research from the past decade on digital technology for promoting adolescent mental health. A systematic literature review and meta-analysis are used to explore which types and features of technology can enhance mental health. We believe that the present study makes a meaningful contribution to scholarship because it is among the earliest to report on the impact of technology-enhanced mental health interventions and has revealed crucial influencing factors that merit careful consideration during both research and practical implementation. The following three research questions guided our systematic review and meta-analysis:

1. What is the current status of global research on digital technology for promoting children and adolescent mental health?
2. What digital technology characteristics support the development of mental health among children and adolescents?
3. How effective is digital technology in promoting the mental health of children and adolescents? What factors have an impact on the effectiveness of digital technology interventions?

## 2 Study 1: systematic literature review

### 2.1 Method

#### 2.1.1 Study design

This study used the systematic literature review method to analyze the relevant literature on the promotion of mental health through digital technology. It followed the Preferred Reporting Items for Systematic Reviews and Meta-Analysis statement for the selection and use of research methods. The protocol for this study was registered with INPLASY (2023120004). Standardized systematic review protocol is used to strictly identify, screen, analyze, and integrate literature (Bearman et al., 2012). To clarify the research issues, systematic literature reviews typically comprise the following six key procedures: planning, literature search, literature assessment, data extraction, data synthesis, and review composition (Lacey and Matheson, 2011).

#### 2.1.2 Literature search

To access high-quality empirical research literature from the past decade, this study selected SCIE and SSCI index datasets from the Web of Science core database and Springer Link. Abstracts containing the English search terms “mental health or psychological health or psychological wellbeing” AND “technology or technological or technologies or digital media” AND “K-12 or teenager or children or adolescents or youth” were retrieved. The search period spanned from January 1, 2013, to July 1, 2023, and 1,032 studies were obtained. To ensure the relevance of the studies to the research question, the relevant inclusion and exclusion criteria were developed based on the 1,032 studies retrieved. The specific criteria are listed in Table 1.

**Table 1:** Literature screening criteria.

Screening criteria		Inclusion	Exclusion
1	Is the document duplicated?	No	Yes
2	Is the study available in full?	Yes	No
3	Is the document a journal article?	Yes	No
4	Is the study a review paper?	No	Yes
5	Is the study topic highly relevant to the promotion of mental health?	Yes	No
6	Does the study consider whether mental health interventions use digital technologies?	Yes	No
7	Are the subjects of the study adolescents (6–18 years old or mean age < 18 years)?	Yes	No

Digital technology refers to the technical means of utilizing computers and electronic devices to acquire, store, transmit, and process information through digitization, encoding, and data processing methods.

**Figure 1:**
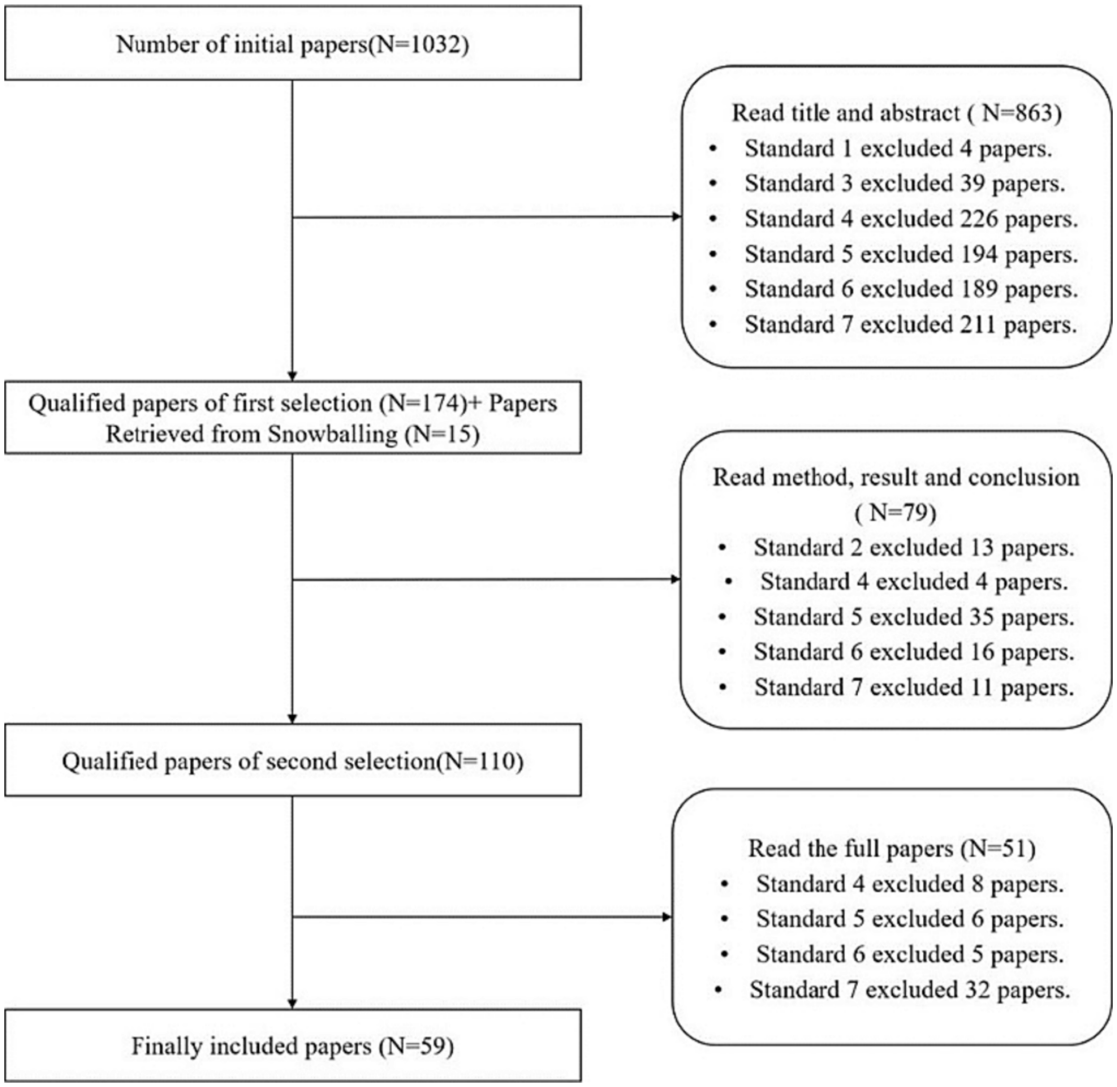
Screening process and results.

In this study, we followed a systematic literature review approach and screened the retrieved studies based on the above selection criteria. We conducted three rounds of screening and supplemented new studies through snowballing, ultimately including 59 effective sample documents. The specific process is shown in Figure 1.

#### 2.1.3 Coding protocol

To extract key information from the included papers, we systematically analyzed 59 studies on the basis of reading the full text. Our coding protocol encompassed the following aspects: (a) basic information about the study, including the first author, publication year, publication region, study type, study object, and intervention duration; (b) the type of technology used in the study, including apps, chatbots, serious games, VR/AR, short messaging service (SMS), telemedicine services, and others; (c) mental health issues, including depression and anxiety, mental illness, bullying, lack of social and emotional competence, mental health issues caused by COVID-19, and other mental health issues; and (d) experimental data (mean, sample size, standard deviation or *p*-value, *t*-value, etc.). By capturing basic study information, we establish a foundation for comparing and contextualizing the selected studies. The type of technology used is crucial as it reflects the innovative approaches and their technical affordances. Mental health issues are the core focus that dictates the objectives of the technological interventions as well as their suitability and relevance. Experimental data provides quantifiable evidence to support the effectiveness claims and lays a foundation for the meta-analysis. Together, these four coding aspects offer a holistic view for a comprehensive understanding and analysis of the existing literature. The document coding was completed jointly by the researchers after confirming the coding rules and details through multiple rounds of negotiation. Problems arising in the coding process were intensively discussed to ensure consistency and accuracy of the coding.

### 2.2 Results and discussion

#### 2.2.1 Study and sample characteristics

**Figure 2:**
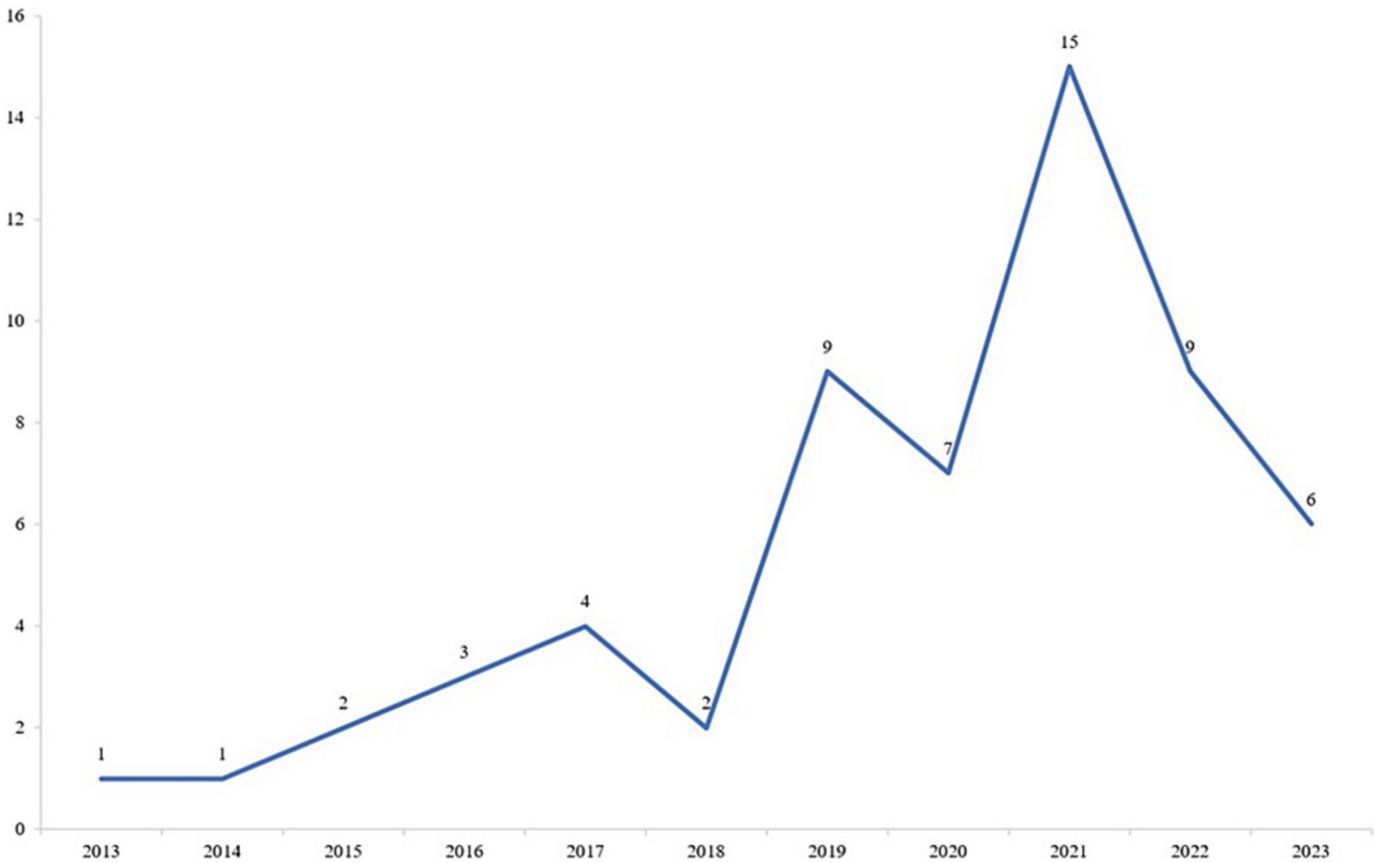
Trend in the number of studies published in the past decade.

As shown in Figure 2, in terms of the time of publication, the number of studies has gradually increased from 2013 to 2021 along with the development of digital technology. The proportion of studies published in the past 5 years (2019–2023) accounted for 76.3% of the total (45/59), with a peak in 2021 with 15 papers. Social isolation, school suspension, and reduced extracurricular activities caused by COVID-19 may exacerbate mental health issues among children and adolescents, which has attracted more researchers to explore the application of digital technology to mental health treatment.

From the perspective of published journals, all of the studies were published in 41 kinds of journals, but two fields were clear leaders: 46 studies (77.97%) were published in medical journals, followed by psychological journals (13.56%). Table 2 shows the source distribution and types of the sample studies. Looking at the country of the first author, the largest number of articles came from the Americas, including the United States and Canada, accounting for 40.7%, followed by European countries, including the United Kingdom and Finland. Only one article came from the African region. In terms of the research types, experimental research was the main type, followed by mixed research, and the number of investigation- and design-based research was relatively small.

**Table 2:** Coding results for sample studies.

Region	Sample size	Empirical research					
		Experimental research	Quantitative research	Qualitative research	Mixed research	Investigation research	Design-based research
Asia	6	4	1	0	1	0	0
Europe	19	11	1	1	2	2	2
America	24	12	3	2	5	1	1
Oceania	9	2	0	3	2	1	1
Africa	1	1	0	0	0	0	0
total	59	30	5	6	10	4	4

Looking more specifically at the research objects, the age range varied from 6 to 18 years. Overall, adolescents aged 13–18 years received more attention, while only six articles considered the younger age group aged 6–12 years. In addition, by coding the sample size of the studies, we found that the quality and size of the studies varied, ranging from small pilot studies or case studies to large-scale cluster studies. For example, Orlowski et al. (2016) conducted a qualitative study on adolescents with experience of seeking help in mental health care institutions in rural Australia; in their study, 10 adolescents with an average age of 18 years were recruited for semi-structured interviews to determine their attitudes and views on the use of technology as a mental health care tool. Another large-scale, randomized controlled trial is planned to enroll 10,000 eighth graders to investigate whether cognitive behavioral therapy (CBT) provided by a smartphone app can prevent depression (Werner-Seidler et al., 2020).

#### 2.2.2 Mental health issues and technology interventions

**Figure 3:**
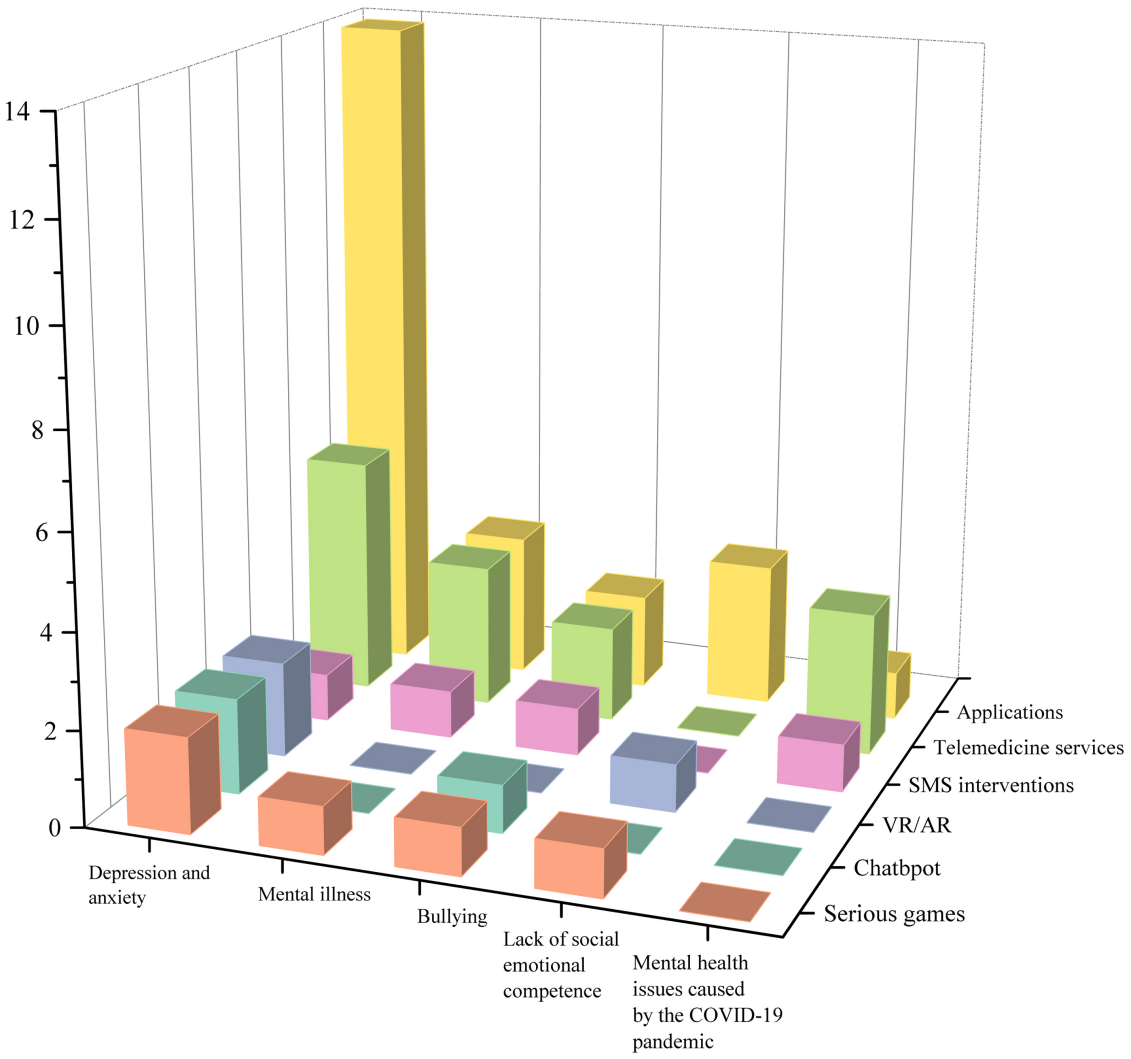
Numbers of studies by mental health issues and technology interventions.

Based on the coding results, we present the total number of studies that correspond to both mental health issues and technological interventions in Figure 3. Our findings indicate that apps represent the most prevalent form of digital technology, particularly in addressing depression and anxiety. Telemedicine services also rank highly in terms of utilization. Contrarily, there are comparatively fewer apps involving virtual reality (VR), augmented reality (AR), chatbots, and serious games. Below, we delve into the specifics of digital technology application and its unique affordances, tailored to distinct mental health issues.

##### 2.2.2.1 Depression and anxiety

Depression and anxiety in adolescents have become increasingly common, and their presence may signal the beginning of long-term mental health issues, with approximately one in five people experiencing a depressive episode before the age of 18 years (Lewinsohn et al., 1993). This has a range of adverse consequences, including social dysfunction, substance abuse, and suicidal tendency. From the 59 articles considered here, 29 studies used digital technology to treat depression- and anxiety-related symptoms in adolescents. Among the many types of digital technology considered, 19 studies used apps or educational websites as intervention tools, accounting for 76%, followed by serious games, chatbots, and VR with two articles each.

Apps are a broad concept, but they typically refer to software that can be downloaded from app stores to mobile devices such as phones or tablets. Due to characteristics such as their clear structure, ease of use, accessibility, strong privacy, interactivity, and multi-modularity, apps and educational websites are commonly used as tools for technological interventions. For example, Gladstone et al. (2015) developed an interactive website called CATCH-IT to prevent depression in adolescents; the site includes 14 optional modules. The course design of each module applies educational design theories, such as attracting learners’ attention, reviewing content, enhancing memory, and maintaining transfer. Apps and websites can also combine CBT with digital technology. The theoretical framework of CBT is rooted in a core assumption that depression is caused and maintained by unhelpful cognitions and behaviors. Treatment thus focuses on improving the function of these areas by applying skill-based behavioral strategies (Wenzel, 2017). Multiple studies have incorporated CBT’s emphasis on reducing cognitive errors and strengthening positive behavior into their designs by, for example, using fictional storylines to help participants correct irrational thought patterns during reflective tasks, thereby improving patients’ depression conditions (Stasiak et al., 2014; Topooco et al., 2019; Neumer et al., 2021).

In addition to the intervention methods involving apps and websites, serious games have also become a prospect for treating depression due to their interesting and interactive characteristics. Low-intensity human support combined with smartphone games may potentially reduce the resource requirements of traditional face-to-face counseling. Games contain complete storylines and competitive and cooperative tasks between peers in the form of levels that encourage adolescents to reflect on quizzes at the end of each challenge (Gonsalves et al., 2019). Game designs tend to use flow theory, which emphasizes the dynamic matching of game challenges and the user’s own skill level (Csikszentmihalyi, 2014). During game design, it is necessary to provide users with an easy-to-use and interesting gaming experience, as well as appropriate difficulty challenges, clear rules and goals, and instant feedback, which will help them relax and relieve stress, concentrate on changing cognitive processes, and improve their mood.

Two articles also consider the use of chatbots in interventions. Chatbots act as a dialog agent (Mariamo et al., 2021), which makes the intervention process more interactive. Establishing a relationship of trust between adolescents and chatbots may also help lead to better results in depression and anxiety treatment. Chatbot functions are typically integrated into apps (Werner-Seidler et al., 2020) and tend to be developed as part of the program rather than as a separate technological tool.

In recent years, with the gradual marketization of head-mounted VR devices, VR technology has been increasingly applied to mental health interventions. Studies have shown that the effectiveness of VR apps is often attributed to the distraction created by immersive environments, which produce an illusion of being in a virtual world, thus reducing users’ awareness of painful stimuli in the real world (Ahmadpour et al., 2020). In the treatment of depression and anxiety for adolescents, active distraction supported by VR can engage users in games or cognitive tasks to redirect their attention to virtual objects and away from negative stimuli. Studies have also shown that, in addition to providing immersion, VR should create a pleasant emotional experience (e.g., the thrill of riding a roller coaster) and embed narrative stories (e.g., adventure and exploration) to meet adolescents’ need for achievement (Ahmadpour et al., 2019).

##### 2.2.2.2 Mental illness

In this study, we define mental illness as neurological developmental problems other than depression and anxiety. Among the 59 reviewed articles, 10 were coded as mental illness, including obsessive-compulsive disorder, attention-deficit/hyperactivity disorder, conduct disorder, oppositional defiant disorder, personality disorder, drug addiction, bipolar disorder, and non-suicidal self-injury. For the treatment of mental illness, mobile apps based on CBT appeared twice in 10 articles, while other technology types included SMS intervention, serious games, remote video conferencing, and mobile sensing technology.

Similar to apps for treating depression and anxiety, adolescent patients believe that the apps have good usability and ease of use and can encourage them to share their thoughts, feelings, and behavioral information more openly and honestly while protecting their privacy (Adams et al., 2021). However, due to the severe condition of patients with mental illness, the apps not only are used independently by patients but also serves as a bridge between therapists and patients. Therapists can thus closely monitor treatment progress through behavioral records, which can provide direct feedback to both patients and therapists (Babiano-Espinosa et al., 2021).

SMS interventions send specific content text messages to patients. As a longitudinal intervention method, it is convenient, easy to operate, and low cost. For example, Owens and Charles (2016) sent text messages to adolescents with non-suicidal self-injury behaviors in an attempt to reduce their self-mutilation behaviors. The ultimate effect seemed to be unsatisfactory, as interventions for adolescents with self-mutilation behaviors may be better applied in schools and adolescents’ service agencies, which can help them control their self-mutilation behaviors in the early stages and prevent such behaviors from escalating.

There are also studies that have designed six serious games based on CBT frameworks to treat typical developmental disorders in adolescents, including attention-deficit/hyperactivity disorder, conduct disorder, and oppositional defiant disorder (Ong et al., 2019). In the safe environment provided by the game world, the research subjects shape the behavior of the characters in the context through rule learning and task repetition, which allows them to master emotional management strategies and problem-solving skills. In addition to interventions, digital technology can also be used to evaluate treatment effectiveness and the type of disease. Orr et al. (2023) used mobile sensing technology and digital phenotyping to quantify people’s behavioral data in real time, thereby allowing diagnosis and evaluation of diseases.

##### 2.2.2.3 Bullying

Bullying generally includes traditional bullying and cyberbullying. Traditional bullying usually manifests as direct physical violence or threats of abuse against victims, as well as indirect methods such as spreading rumors and social exclusion. Cyberbullying is defined as intentional harm to others through computers, mobile phones, and other electronic devices. Data show that, as of 2021, the proportion of adolescents who have experienced cyberbullying in the United States may be as high as 45.5% (Patchin, 2021), which indicates that it has become a serious social problem. Among the nine articles on the topic of bullying and cyberbullying, three used SMS intervention methods, and two used mobile apps; chatbots, technology-supported courses, and CBT-based telemedicine services were also used in the mental health treatment for patients who had been bullied and cyberbullied.

The SMS intervention for bullying implemented personalized customization, and the automatic SMS content can be customized based on the subjects’ previous questionnaire or completed self-report status (Ranney et al., 2019). The subjects are required to rate their feelings at the end of the day and report whether they were bullied that day. The psychotherapist then made adjustments based on their actual situation, and if necessary, the psychotherapist would also contact specific subjects to provide offline psychological counseling services (Ranney et al., 2019). In addition to having similar functions as the SMS intervention (Kutok et al., 2021), mobile apps can provide opportunities for personalized learning, where a variety of learning methods can be applied (e.g., providing therapist guidance, conducting meetings, and conducting family practice activities) to promote the acquisition of mental health skills (Davidson et al., 2019). Furthermore, for adolescents, touchscreen learning, interactive games, and video demonstrations can enhance their enthusiasm for participating in the treatment process.

Chatbots with specific names and images were also used to guide research subjects through a series of online tasks in the form of conversations, including watching videos involving bullying and cyberbullying among adolescents, provoking self-reflection through questions and suggestions, and providing constructive strategic advice (Gabrielli et al., 2020). Digital technology-supported courses and CBT-based telemedicine services both make full use of the convenience of technology, effectively addressing the time- and location-based limitations of traditional face-to-face treatment. Digital courses can be implemented on a large scale in schools through teacher training, and compared with professional medical services, such courses have a wider target audience and can play a scientific and preventive role in bullying and cyberbullying. Telemedicine services refer to the use of remote communication technology to provide psychological services (Joint Task Force for the Development of Telepsychology Guidelines for Psychologists, 2013). For families with severely troubled adolescents, telemedicine allows parents and children to meet together, increasing the flexibility of timing, and one-on-one video services can help to build a closer relationship between patients and therapists.

##### 2.2.2.4 Lack of social emotional competence

In research, social emotional competence typically refers to the development of emotional intelligence in adolescents (De la Barrera et al., 2021), which also includes personal abilities (self-awareness and self-management), interpersonal relationships (social awareness and interpersonal skills), and cognitive abilities (responsible decision-making) (Collaborative for Academic, Social, and Emotional Learning, 2020). It is an important indicator for measuring the mental health level of adolescents. People with positive social emotional intelligence are less likely to experience mental health issues such as depression, anxiety, and behavioral disorders. Using digital technology to promote social emotional development is becoming increasingly common, and in six intervention studies on social emotional competence, apps, serious games, VR technology, and SMS interventions were used.

The studies considered all emphasized the importance of interactive design in digital technology to enhance social and emotional skills, as interactive technology can increase students’ engagement, resulting in positive learning experiences. For example, Cherewick et al. (2021) designed a smartphone app that can be embedded with multimedia learning materials, allowing adolescents to watch social and emotional skill–related learning videos autonomously and complete topic reflection activities with family/peers after school. The app also has rich teaching interaction functions, allowing teachers to evaluate and share course and learning materials, which can provide pleasant learning experiences to students while also improving the flexibility of teaching. In addition to teacher–student interaction, another paper also mentioned the importance of human–computer interaction for developing social emotional competence. The fun and interactivity of the app are the key to attracting adolescents to download and use it, and it can also have a positive effect on improving students’ self-management and decision-making skills (Kenny et al., 2016).

Unlike the treatment of depression and anxiety, the application of VR in the cultivation of social emotional competence not only relies on its highly immersive characteristics but also emphasizes the positive effects of multi-sensory experiences on emotional regulation. By utilizing various sensor devices and visualization devices, adolescents are provided with ideal visual, auditory, and tactile guidance and regulation, which can enhance their emotional regulation abilities and relieve psychological stress (Wu et al., 2022). Existing studies have integrated dance and music into virtual scenes (Liu et al., 2021), using virtual harmonic music therapy to allow users to relax physically and mentally while enjoying music, thereby reducing stress and anxiety. VR technology is also highly adaptable and generalizable, which can help in building diverse scenes that meet the psychological expectations of patients based on the characteristics of the different treatment objects.

##### 2.2.2.5 Mental health issues caused by the COVID-19 pandemic

The global outbreak of COVID-19 created severe challenges for the mental health of adolescents. Factors such as lack of social contact, lack of personal space at home, separation from parents and relatives, and concerns about academics and the future have exacerbated mental health risks, leading to increased loneliness, pain, social isolation, mental disorders, and symptoms of anxiety, depression, and stress. The reports from five studies indicated that the COVID-19 pandemic has exacerbated mental health issues in adolescents. During the pandemic, technology—which is not limited by time and space—became the preferred method of treatment. Apps, remote health services, and online training courses were used in research. The apps were resource-oriented and evidence-based interventions that allowed patients to interact with therapists through remote conferencing and encouraged patients to self-reflect and express themselves after the conference to improve their mental condition (Gómez-Restrepo et al., 2022). Remote health services combined CBT and dialectical behavior therapy with professional counselors engaging in online communication with patients for several weeks. This was in line with research that indicates that the establishment of a positive relationship between therapists and patients is the foundation for obtaining good effect (Zepeda et al., 2021).

##### 2.2.2.6 Other mental health issues

In addition to the common mental health issues mentioned above, there were also interventions mentioned in the literature for improving body image anxiety, mental issues caused by hospitalization, and reading disabilities through digital technology means. Due to its high-immersion and simulation characteristics, VR technology was selected for improving mental health issues such as loneliness, disconnection from peers, and academic anxiety caused by hospitalization (Thabrew et al., 2022). Immersive VR experience technology used 360° panoramic live broadcast and VR headphones to enable hospitalized adolescents to indirectly participate in social activities through cameras in school or home environments, as well as to contact peers and teachers through methods such as text messages; such interventions are conducive to improving social inclusion, social connectivity, and happiness. Furthermore, two studies mentioned body image anxiety, especially targeting female audiences, and the research integrated body image CBT techniques into serious games and chatbots (Mariamo et al., 2021; Matheson et al., 2021), using interesting interactive exploration and free dialog forms to help adolescents gain a correct understanding of body image and solve body image anxiety issues.

Another study used eye-tracking technology to treat children with reading disabilities (Davidson et al., 2019). The researcher developed a reading evaluation platform called Lexplore, which used eye-tracking technology to monitor children’s eye movements when reading to determine the cognitive processes behind each child’s individual reading style and then design appropriate strategies to improve their reading difficulties.

## 3 Study 2: meta-analysis

### 3.1 Method

To explore the effect of digital technology in promoting mental health, this study used a meta-analysis to assess 10 papers. It includes both experimental and quasi-experimental research studies. CMA3.0 (Comprehensive Meta-Analysis 3.0) was used, and the meta-analysis process consisted of five phases.

Phase 1: Literature screening, based on the prior stage of literature information coding. Relevant literature was filtered using the following criteria for meta-analysis: (a) the study must compare “technical intervention” and “traditional intervention”; (b) the study should report complete data that can generate the effect amount (e.g., average, sample size, standard deviation or *t*-values, *p*-values, etc.); and (c) the dependent variables in the study should contain at least one aspect of mental health.

Phase 2: Effect size calculation. In the case of a large sample size, there is little difference between Cohen’s d, Glass, and Hedges’ *g* values, but Cohen’s d can significantly overestimate the effect size for studies with a small sample (Hedges, 1981). Therefore, Hedges’ g was used as the effect size indicator in this study.

Phase 3: Model selection. Meta-analyses include fixed- and random-effects models. Different models may produce different effect sizes. Due to the differences in sample size, experimental procedures, and methods among the initial studies included in the meta-analysis, the estimated average effect values may not be completely consistent with the true population effect values, which results in sample heterogeneity. This study used the method proposed by Borenstein et al. (2009) to establish fixed- and random-effects models to eliminate the influence of sample heterogeneity. When the heterogeneity test (*Q* value) results were significant, the random-effects model was used; otherwise, the fixed-effects model was used.

Phase 4: Testing of main effects and moderating effects. Based on the selected model, a test of the main effects was conducted. Meanwhile, if heterogeneity was present, a test of moderating effects could be conducted.

Phase 5: Publication bias test. Publication bias is a common systematic error in meta-analyses and refers to a tendency for significantly significant research results to be more likely to be published than non-significant results. This study used a funnel plot to visually assess publication bias qualitatively and then further quantitatively assessed publication bias using Begg’s rank correlation method and the trim and fill method.

### 3.2 Results and discussion

#### 3.2.1 Inclusion and coding results

For the studies that met the requirements of the meta-analysis, detailed classification was carried out based on the following variables one-by-one on the basis of the systematic review coding: (a) basic information (authors, year, sample size); (b) age stage, which is divided into three categories: primary school, junior high school, and senior high school; (c) mental health issues, including depression, bullying, and mental health issues caused by COVID-19; (d) technology type, including app, telemedicine, and chatbots; (e) intervention duration, coded as short-term for interventions less than a month and long-term for intervention that lasted more than a month; and (f) effect size. The coding results are shown in Table 3.

**Table 3:** Research coding results included in meta-analysis.

Research	Sample size	Age stage	Mental health problem	Technology type	Intervention duration	g (95% CI)
Anttila2021	82	J	D	A	LI	0.045
Jones2020	35	S	D	A	LI	0.192
kor2023	1,670	J	M	A	LI	0.142
nicoll2022	17	S	D	C	SI	0.005
Pavarini2023	100	S	M	T	SI	1.316
Ruggiero2015	2,000	S	D	R	LI	0.192
Stasiak2014	34	S	D	A	LI	0.52
Stewart2017	15	P	B	T	SI	0.786
Topooco2019	70	S	D	T	LI	0.893
Zepeda2023	27	J	C	T	SI	0.379

A, App software; B, Bullying (including cyberbullying, school bullying); C, Chatbot; M, Mental health issues caused by COVID-19; D, Depression; J, Junior high school; P, Primary school; S, Senior high school; T, Telemedicine; LI, Long-term intervention; SI, Short-term intervention.

#### 3.2.2 The overall effect of digital technology on mental health outcomes

According to the results of the heterogeneity test in Table 4, the Q test is significant (*p* < 0.001), which indicates that there is significant heterogeneity among the samples. The random-effects model was therefore selected as the more reasonable option. The pooled effect size is 0.43. According to the criteria proposed by Cohen (1992), 0.2, 0.5, and 0.8 are considered the boundaries of small, medium, and large effect sizes, respectively. It can be seen that the effect size for the promotion of mental health by digital technology is moderate and significant. At the same time, the lower limit of the 95% confidence interval is greater than 0 for each study, which indicates that the probability of the effect size being caused by chance is very small. In addition, the *I*^2^ value is 78.164, which indicates that the heterogeneity between studies is high. Important moderating variables therefore may exist (Higgins and Green, 2008), and additional moderating effect tests need to be conducted.

**Table 4:** Overall effect of technology on mental health.

Model	*N*	Hedges’ g	95% CI	Heterogeneity test			
				*Q*	df	*p*	*I*^2^
Fixed model	4,050	0.231^***^	0.154–0.308	41.216	9	<0.001	78.164
Random model		0.430^***^	0.198–0.661				

The significance level of 0.05 is adopted in all analyses.

#### 3.2.3 Moderating effect test

Moderating effect tests were conducted on four variables: age stage, mental health issues, technology type, and intervention duration. As shown in Table 5, among the four moderating variables, only the age stage has a significant moderating effect (*p* < 0.05). In particular, the effect size is the largest for the primary school stage, followed by the senior high school stage with a moderate promoting effect. In addition, although the effect size for the junior high school stage is small, it is still significant, which may be related to the limited number of studies considering this population. The results also indicate that the moderating effects of mental health issues, technology type, and intervention duration are not significant. However, it can be seen that digital technology methods have the largest effect size for treating psychological problems caused by COVID-19, while compared with apps and chatbots, remote medical services can achieve better effects. In terms of treatment duration, the effect size for short-term interventions is greater than that for long-term interventions.

**Table 5:** Regulatory effect test of technology (random-effect model).

Variable	Category	*k*	*N*	*g*	95% CI	Heterogeneity		
						*Q*	df	*p*
Age stage	Primary school	1	15	0.786	0.231–0381^**^	7.935	2	0.019^*^
	Junior high school	3	1,779	0.151	0.060–0.242^*^			
	Senior high school	6	2,256	0.539	0.131–0.946^*^			
Mental health issues	Depression	6	2,238	0.290	0.058–0.522^*^	3.023	2	0.221
	Mental health issues caused by COVID-19	3	1,797	0.588	−0.059–1.235			
	Bullying	1	15	0.290	0.244–1.418^**^			
Technology type	Apps software	4	1,821	0.148	0.059–0.237^*^	5.791	2	0.055
	Telemedicine	5	2,212	0.690	0.253–1.127^**^			
	Chatbot	1	17	0.005	−0.912–0.921			
Intervention duration	Short-term intervention	4	159	0.681	0.138–1.224^*^	2.280	2	0.131
	Long-term intervention	6	3,891	0.242	0.071–0.414^**^			

#### 3.2.4 Publication bias test

**Figure 4:**
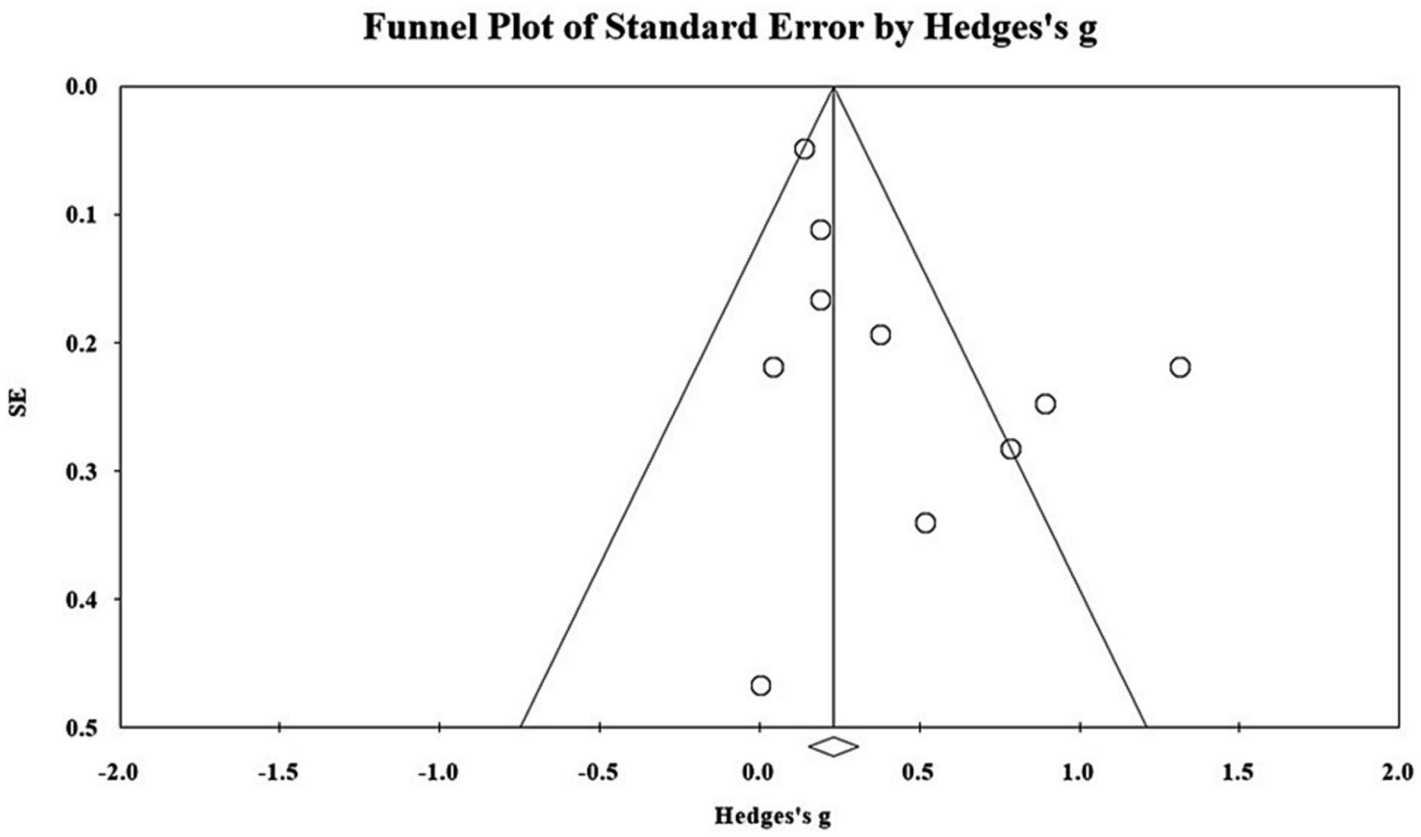
Distributions of effect sizes for mental health treatment outcomes.

This study used funnel plots, Begg’s test, and the trim and fill method for the publication bias test. As shown in Figure 4, the distribution of effect values in the study shows uneven and asymmetric distribution on both sides of the mean effect value, which initially suggests the possibility of publication bias. Begg’s test was thus used for further testing. Begg’s test is a method of quantitatively identifying bias using a rank correlation test, and it applies to studies with a small sample. The result of Begg’s test shows that *t* = 0.267, *p* = 0.283, *Z* = 1.01 < 1.96, which indicates that there is no obvious publication bias. Finally, the censoring method was used to censor the literature on both sides of the effect value, and this revealed that the effect value was still significant. In summary, there is negligible publication bias.

## 4 Conclusion and implications

### 4.1 Summary of key findings

This study made a systematic review and meta-analysis of 59 studies on digital technology promoting adolescents’ mental health over the past decade. Based on the investigation of current research, the types and characteristics of the commonly used technology interventions for different mental health issues were analyzed, and the actual effects and potential regulatory variables of digital technology in promoting mental health were investigated in the meta-analysis. The main findings are outlined below.

- Over the past decade, especially between 2013 and 2021, the number of studies on digital technology promoting adolescents’ mental health has generally shown an upward trend, with nearly 80% of the literature being published in medical journals.
- Digital technology is most commonly used to intervene in the mental health issues of adolescents aged 13–18 years, and children in the younger age group (6–12 years old) receive relatively less attention.
- Depression and anxiety disorders are the mental health issues that received the most research attention, followed by obsessive-compulsive disorder, attention-deficit hyperactivity disorder, conduct disorder, and other mental illnesses. There were also studies on, in decreasing order of the number of studies, bullying, social emotional competence deficiency, and mental health issues caused by COVID-19, dyslexia, and adolescent body image anxiety.
- Apps with convenience, ease of use, interactivity, and remote communication were most commonly used to treat mental health issues. Serious games, remote health services, and text message intervention were less often used, and only three studies used VR, which is difficult to realize for mental health treatment.
- Digital technology plays a significant role in promoting the treatment of mental health issues of adolescents, especially in primary and senior high school.

### 4.2 Interpretation and insights

The findings of this study highlight the nuanced role played by digital technology in promoting mental health for children and adolescents. While technology has broadened the scope of mental health interventions with innovative apps and programs, it should be viewed as a complement to traditional face-to-face approaches, not a replacement (Aguilera, 2015), as they cannot replicate the personal connection and empathy provided by a trained mental health professional. Moreover, different technologies vary in effectiveness for specific mental health issues, emphasizing the need for careful evaluation of their benefits and limitations. For instance, virtual reality, cognitive behavioral therapy apps, and online support platforms have shown promise for in addressing depression and anxiety, but their effects vary depending on individual needs and contexts, suggesting the non-uniform efficacy of digital technologies across mental health conditions.

Furthermore, this study also draws attention to the limited incorporation of digital technology in mental health education, especially among children aged 6 to 12. Given the significance of this developmental stage, where emotional management, relationships, and mental health knowledge are crucial, innovative digital approaches that draw upon the unique affordances of mobile apps, online courses, and virtual reality are warranted to deliver interactive and personalized learning experiences. Nevertheless, this innovation poses challenges and risks, including addiction to virtual environments and a reduction in social activities, which can also negatively impact the mental health of youth (Taylor et al., 2020). Therefore, striking a balance between harnessing technology’s potential and mitigating its risks is essential, emphasizing the need for responsible and targeted use of digital tools in mental healthcare and education.

### 4.3 Implication for practice and future research

Based on the results of the systematic review and meta-analysis, this study puts forward the relevant implications for practice and research. First, for mental health education service personnel, we suggest that the first step is to fully utilize the characteristics of digital technology and select the most appropriate digital intervention tools for different mental health issues. For example, apps are more suitable for the treatment of depression, anxiety, and mental illnesses. When facing adolescents who have been bullied, text message interventions may be a good choice. In addition, serious games and VR could play a greater role in developing adolescents’ social emotional competence.

Second, for mental health counselors or school mental health workers, it is necessary to consider learner characteristics and intervention duration, among other factors. In contrast to previous research results (Tang et al., 2019), we found that the regulating effect of age was significant, so therapists need to implement personalized technical interventions for adolescents at different age stages. Short-term interventions seem to induce a greater effect size, so lengthy interventions should be avoided, as they are more likely to cause marginal effect and develop technical immunity for the youth population.

Third, for technology intervention developers, it is important to recognize that not all practitioners (e.g., psychologists, therapists) are technology savvy. In the process of designing mental health apps and VR interventions, it is necessary to provide sufficient technical support, such as instructional manuals and tutorial videos, to reduce the potential digital divide. It is also essential to arrange for appropriate technical personnel to provide safeguard services and training continuously, ensuring the personal safety and cybersecurity of practitioners and patients during intervention sessions.

For researchers, we suggest that, first, more empirical studies are needed to report first-hand experimental results. Most of the existing studies only described the experimental scheme and lacked key research results. It is hoped that future research will report the results as comprehensively as possible to improve the credibility and reliability of meta-analytical results. Second, the number of studies on moderating effects in the meta-analysis was relatively small. For example, there was only one study on the primary school population. Future research needs to focus on people who have paid less attention to existing studies and thus enhance the understanding of technology interventions in mental health. Finally, there have been few studies that analyze cost-effectiveness, which is key to determining whether technical interventions can be normalized and sustainable. Future studies need to conduct sufficient investigation and report on the cost-effectiveness of digital technology interventions, including the development and maintenance costs of VR (Kraft, 2020).

## Data availability statement

The raw data supporting the conclusions of this article will be made available by the authors, without undue reservation.

## Author contributions

TC: Data curation, Formal analysis, Investigation, Visualization, Writing – original draft. JO: Formal analysis, Investigation, Writing – original draft. GL: Formal analysis, Writing – review & editing. HL: Conceptualization, Methodology, Supervision, Writing – review & editing.
